# A Cross-Sectional Survey on Medication Management Practices for Noncommunicable Diseases in Europe During the Second Wave of the COVID-19 Pandemic

**DOI:** 10.3389/fphar.2021.685696

**Published:** 2021-06-07

**Authors:** Tamás Ágh, Job FM van Boven, Björn Wettermark, Enrica Menditto, Hilary Pinnock, Ioanna Tsiligianni, Guenka Petrova, Ines Potočnjak, Fatjona Kamberi, Przemyslaw Kardas

**Affiliations:** ^1^ Syreon Research Institute, Budapest, Hungary; ^2^ Department of Clinical Pharmacy and Pharmacology, Medication Adherence Expertise Center of the Northern Netherlands, University Medical Center Groningen, University of Groningen, Groningen, Netherlands; ^3^ Department of Pharmacy, Faculty of Pharmacy, Uppsala University, Uppsala, Sweden; ^4^ Faculty of Medicine, Vilnius University, Vilnius, Lithuania; ^5^ Department of Pharmacy, CIRFF, Center of Pharmacoeconomics and Drug Utilization Research, University of Naples Federico II, Naples, Italy; ^6^ Usher Institute, University of Edinburgh, Edinburgh, United Kingdom; ^7^ Department of Social Medicine, School of Medicine, University of Crete, Crete, Greece; ^8^ Departement of Social Pharmacy and Pharmacoeconomics, Faculty of Pharmacy, Medical University of Sofia, Sofia, Bulgaria; ^9^ Institute for Clinical Medical Research and Education, University Hospital Centre Sisters of Charity, Zagreb, Croatia; ^10^ Faculty of Health, Research Center of Public Health, University of Vlore "Ismail Qemali", Vlore, Albania; ^11^ Medication Adherence Research Centre, Department of Family Medicine, Medical University of Lodz, Lodz, Poland

**Keywords:** noncommunicable diseases, drug therapy, persistence, medication adherence, COVID-19, pandemic, healthcare system, medication management

## Abstract

Maintaining healthcare for noncommunicable diseases (NCDs) is particularly important during the COVID-19 pandemic; however, diversion of resources to acute care, and physical distancing restrictions markedly affected management of NCDs. We aimed to assess the medication management practices in place for NCDs during the second wave of the COVID-19 pandemic across European countries. In December 2020, the European Network to Advance Best practices & technoLogy on medication adherencE (ENABLE) conducted a cross-sectional, web-based survey in 38 European and one non-European countries. Besides descriptive statistics of responses, nonparametric tests and generalized linear models were used to evaluate the impact on available NCD services of the number of COVID-19 cases and deaths per 100,000 inhabitants, and gross domestic product (GDP) per capita. Fifty-three collaborators from 39 countries completed the survey. In 35 (90%) countries face-to-face primary-care, and out-patient consultations were reduced during the COVID-19 pandemic. The mean ± SD number of available forms of teleconsultation services in the public healthcare system was 3 ± 1.3. Electronic prescriptions were available in 36 (92%) countries. Online ordering and home delivery of prescription medication (avoiding pharmacy visits) were available in 18 (46%) and 26 (67%) countries, respectively. In 20 (51%) countries our respondents were unaware of any national guidelines regarding maintaining medication availability for NCDs, nor advice for patients on how to ensure access to medication and adherence during the pandemic. Our results point to an urgent need for a paradigm shift in NCD-related healthcare services to assure the maintenance of chronic pharmacological treatments during COVID-19 outbreaks, as well as possible future disasters.

## Introduction

Noncommunicable diseases (NCDs) are a major public health issue, responsible for 80% of all years lived with disability and 70% of all deaths worldwide [World Health Organization (WHO), 2018a; Institute for Health Metrics and Evaluation (IHME), 2020]. However, because of the COVID-19 pandemic [World Health Organization (WHO), 2020a], NCDs have not been the main priority for healthcare services during the last year, as COVID-19 infected more than 114 million individuals, with 2.5 million deaths worldwide [European Centre for Disease Prevention and Control (ECDC), 2021].

Notably, the epidemics of COVID-19 and NCDs are closely interlinked. Patients with NCDs are more susceptible to severe and fatal COVID-19 infection (Clark et al., 2020; CDC COVID-19 Response Team, 2020; Noor and Islam, 2020; Sheldon and Wright, 2020). Conversely, COVID-19 negatively affects lifestyle habits (e.g., lower physical activity, increased tobacco use); indirectly increasing the risk of developing and progression of NCDs (Palmer et al., 2020; Papachristou et al., 2020). There is also a growing body of evidence that effective management of NCDs has a protective effect against COVID-19 infection, and its severity. For example, continuous statin use during the month prior to hospital admission for COVID-19 was associated with a lower risk of developing severe infection, and a faster time to recovery (Daniels et al., 2020).

Although, the appropriate management of NCDs is important during the COVID-19 pandemic, the intensive focus on COVID-19 prevention and treatment, the lockdown and physical distancing restrictions affected NCD-related healthcare services adversely. In May 2020, World Health Organization (WHO) surveyed service delivery for NCDs across 155 countries [World Health Organization (WHO), 2020b] and found that COVID-19 markedly affected NCD services in all regions and income groups. In three-quarters of countries there was considerable disruption to NCD services (e.g., rehabilitation services, hypertension management, diabetes care, asthma services). To overcome disruption many countries adopted strategies such as telemedicine services to replace in-person consultations (61% of countries) or triage to identify priorities (64% of countries).

The continuity of medication therapy is a cornerstone for the effective management of NCDs (Böhm et al., 2013; Kluge et al., 2020). Even before the COVID-19 pandemic, about 50% of people with long-term conditions were non-adherent to their medication [World Health Organization (WHO), 2003] with potential serious health consequences for individuals (Chowdhury et al., 2013). For example, non-adherence to endocrine therapies in breast cancer patients increases the risk of metastases, disease recurrence and mortality (Font et al., 2019; Lee et al., 2019). Assuring continuous access to medication is a prerequisite of appropriate adherence, which may be compromised by disruptions of healthcare services and physical distancing restrictions due to COVID-19 crisis.

The European Network to Advance Best practices & technoLogy on medication adherencE (ENABLE; CA19132) is a 4-years research initiative across Europe funded by the European Cooperation in Science and Technology (COST) Action. ENABLE brings together researchers from 39 countries, among others, with the objective to evaluate current practices related to medication adherence. ENABLE thus considered it important to survey its members on medication management practices during the pandemic, following deepening concerns that European patients with NCDs may be not receiving appropriate care or access to essential medicines [World Health Organization (WHO), 2020b; Minghui et al., 2020] The aim of this study was to assess and critically evaluate the medication management practices in place for NCDs during the second wave of the COVID-19 pandemic across European countries to inform future pandemic management.

## Materials and Methods

### Study Design

A cross-sectional, web-based survey was carried out including all the members of ENABLE (i.e., healthcare providers and academics with medical or pharmaceutical backgrounds) across 38 European countries (i.e., Albania, Austria, Belgium, Bosnia and Herzegovina, Bulgaria, Croatia, Cyprus, Czechia, Denmark, Estonia, Finland, France, Germany, Greece, Hungary, Iceland, Ireland, Italy, Latvia, Lithuania, Luxemburg, Malta, Moldova, Montenegro, the Netherlands, North Macedonia, Norway, Poland, Portugal, Romania, Serbia, Slovakia, Slovenia, Spain, Sweden, Switzerland, Turkey and the United Kingdom) and one non-European (i.e., Israel) country. The primary outcome of the survey was a better understanding on the medication management practices for NCDs during the second wave of COVID-19 across Europe. Ethical approval was not sought as all participants were ENABLE colleagues reporting publicly available information about their country healthcare systems. No personal data were stored in relation to this study. All respondents were asked if they wished to be acknowledged in publications. This study was reported according to the Checklist for Reporting Results of Internet E-Surveys (CHERRIES) (Eysenbach, 2004).

### Questionnaire Development and Data Collection

**FIGURE 1:**
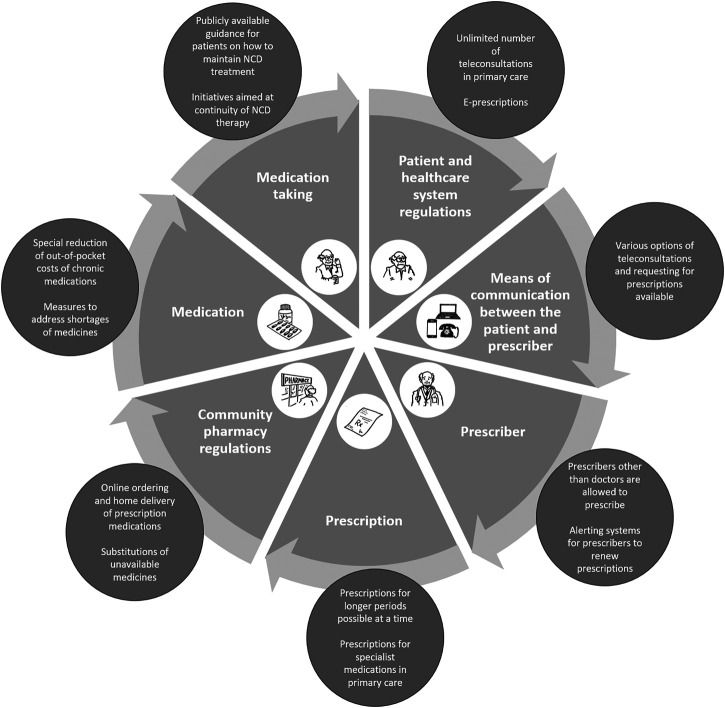
Medication management cycle and interventions that could help continuous pharmacological management of non-communicable diseases.

First, key elements of the medication management cycle of NCDs were defined by the working group as a result of extensive discussions (Figure 1). The questionnaire was developed based upon this framework using following domains: 1) patient and healthcare system regulations, 2) means of communication between the patient and prescriber, 3) prescriber, 4) prescription, 5) community pharmacy regulations, 6) medication, and 7) medication taking. A number of questions was generated for each domain. As part of the validation of the draft questionnaire, seven external experts were asked to assess each item individually with respect to the content, construct and criterion validity and to offer their opinions on the overall questionnaire and on the technical functionality of the electronic questionnaire. Following revision, the final version of the questionnaire contained 33 questions. The majority of questions were closed with responses: “Yes”/“Partly”/“No”/“Do not know.” Respondents were instructed to answer “Yes” if the scenario took place in >80% of cases in the given country, and “Partly” or “No” in 20–80% of cases, and <20% of cases, respectively. The case ratio for the “Partly” answer was determined based on a consensus discussion by ENABLE collaborators and was approved by the external experts.

The link to the validated, web-based survey was sent by email to all ENABLE collaborators (n = 92, two or three representatives from each of 39 countries) on December 15th, 2020. The invitation email informed participants about the purpose of the study, the use and storage of the data and the length of the survey. The survey was posted on SurveyMonkey.com (www. surveymonkey.com). The online questionnaire was distributed over 12 pages, with one to five items per page. A copy of the survey can be seen in Supplementary Figure S1. The average time required to answer the survey was been estimated at 10 min and the respondents were able to review and change their answers before submitting the survey. This voluntary study was sent personally to ENABLE colleagues so that access to the questionnaire by non-invited individuals was unlikely. No incentives were offered to ENABLE collaborators for completing the survey. Respondents were instructed to represent the national perspective, rather than regional, local or their own perspective; and to provide responses as of December 2020. The responses should reflect the current measures in place to support treatment of NCDs in the country, regardless of whether they had been introduced following the COVID-19 outbreak or had been in place before the pandemic. The survey was open until December 29th, 2020, reminders were sent weekly to all participants.

To prevent multiple entries from the same individual, respondents’ names were stored temporarily with the submitted survey. However, unique user identifiers were eliminated before data analysis.

### Data Analysis

A completeness check was conducted after the questionnaire was submitted and only complete questionnaires (regardless of the time needed for filling the questionnaire) were analyzed. Where more than one complete response was received per country, the responses were aggregated. In the few instances where answers differed, respondents were contacted to resolve the inconsistencies prior to data analysis.

In the first step of data analysis, descriptive statistics of the responses were computed. In the next step, we evaluated the impact of three variables on the solutions available to facilitate continuity of medication for NCDs during the COVID-19 pandemic. European Centre for Disease Prevention and Control (ECDC) data were used for the number of COVID-19 cases per 100,000 inhabitants, and the number of COVID-19 deaths per 100,000 inhabitants in 2020 [European Centre for Disease Prevention and Control (ECDC), 2021]. Country income was assessed from World Bank data on gross domestic product (GDP) per capita at purchasing power parity in current international USD (data from 2019) (World Bank, 2021) (Table 1). Differences in the number of COVID-19 cases per 100,000 inhabitants [European Centre for Disease Prevention and Control (ECDC), 2021], the number of COVID-19 deaths per 100,000 inhabitants [European Centre for Disease Prevention and Control (ECDC), 2021], and GDP per capita data (World Bank, 2021) between “Yes”/“Partly”/“No”/“Do not know” response groups per each question of the survey were assessed using Kruskal-Wallis test. A significant Kruskal-Wallis test was followed up by Wilcoxon’s test to calculate pairwise comparisons between response levels with corrections for multiple testing. Poisson generalized linear models with log link using robust standard errors were used to assess the association of the number of teleconsultation approaches (“Yes” and “Partly” response groups were pooled) with the number of COVID-19 cases per 100,000 inhabitants [European Centre for Disease Prevention and Control (ECDC), 2021], the number of COVID-19 deaths per 100,000 inhabitants [European Centre for Disease Prevention and Control (ECDC), 2021], and GDP per capita data (World Bank, 2021). The same analysis was performed for the number of methods for requesting prescriptions for chronic medications (“Yes” and “Partly” response groups were pooled). In all analyses, *p* values of <0.05 were considered statistically significant. Data management and all statistical analyses were performed using R software (The R Foundation for Statistical Computing, Vienna, Austria; version 4.0.2). The raw data supporting the conclusions of this article will be made available by the authors on request, without undue reservation, to any qualified researcher.

**TABLE 1:** Country specific population size, GDP per capita and COVID-19 burden data.

Country	Population size, 2020 [Eurostat (2021); European Centre for Disease Prevention and Control (ECDC) (2021)]	GDP per capita, PPP, 2019 (current international USD) [Word Bank (2021)]	N of COVID-19 cases per 100,000 inhabitants in 2020 [European Centre for Disease Prevention and Control (ECDC) (2021)]	N of COVID-19 deaths per 100,000 inhabitants in 2020 [European Centre for Disease Prevention and Control (ECDC) (2021)]
Albania	2,862,427	14,648.3	2,076	42
Austria	8,858,775	60,418.0	4,115	71
Belgium	11,455,519	56,348.5	5,681	172
Bosnia and Herzegovina	3,280,815	16,288.8	3,433	126
Bulgaria	7,000,039	25,312.1	2,901	110
Croatia	4,076,246	31,130.8	5,224	100
Cyprus	875,899	41,254.4	2,737	15
Czechia	10,649,800	44,295.9	7,012	113
Denmark	5,806,081	62,089.9	2,906	24
Estonia	1,324,820	39,986.2	2,228	19
Finland	5,517,919	53,171.6	669	10
France	67,012,883	50,993.0	3,963	97
Germany	83,019,213	57,530.3	2,139	42
Greece	10,724,599	32,506.4	1,306	46
Hungary	9,772,756	34,966.3	3,365	102
Iceland	356,991	60,132.5	1,612	8
Ireland	4,904,240	89,683.6	2,078	46
Israel	8,655,541	42,897.8	5,109	40
Italy	60,359,546	45,722.6	3,571	125
Latvia	1,919,968	33,020.8	2,213	35
Lithuania	2,794,184	40,016.3	5,297	49
Luxembourg	613,894	124,590.5	7,643	82
Malta	493,559	47,578.2	2,651	45
Moldova	4,033,963	13,627.0	3,616	75
Montenegro	622,182	24,035.9	7,907	111
Netherlands	17,282,163	61,285.0	4,746	67
North Macedonia	2,077,132	18,107.8	4,045	122
Norway	5,328,212	70,005.9	952	8
Poland	37,972,812	35,165.2	3,484	77
Portugal	10,276,617	37,918.4	4,158	69
Romania	19,414,458	33,339.9	3,299	82
Serbia	6,963,764	19,495.1	4,938	48
Slovakia	5,450,421	33,515.9	3,451	46
Slovenia	2,080,908	42,431.2	6,048	139
Spain	46,937,060	43,495.9	4,173	109
Sweden	10,230,185	56,632.1	4,592	88
Switzerland	8,544,527	72,376.0	5,380	85
Turkey	82,003,882	28,133.1	1,729	26
United Kingdom	66,647,112	49,931.6	3,983	113

GDP: gross domestic product; PPP: purchasing power parity.

## Results

A total of 53 (58%) of the 92 ENABLE collaborators from 39 countries (12 countries with more than one respondents) completed the survey. Two-thirds (n = 39) of participants’ professional background was academia (i.e., medical or pharmaceutical sciences) and half of respondents (n = 26) had more than 20 years of work experience.

Our results are reported according to the seven key domains of the medication management cycle of NCDs (Figure 1). Country-specific responses for each item of the survey questionnaire and the detailed results of the statistical analysis can be found in Supplementary Figures S2–S9 and Supplementary Tables S1–S7, respectively.

### Patient and Healthcare System Regulations

All the included countries have public healthcare systems available to all citizens. Consultations in primary healthcare and other ambulatory care are fully or partially covered in all the countries. In 10 (26%) countries there were restrictions in number of consultations without fee in primary care for patients with NCDs. In these countries the mean number of COVID-19 cases per 100,000 inhabitants was significantly lower compared to countries with unlimited free primary care consultations (*p* = 0.017) (Table 2).

**TABLE 2:** Significant results of the statistical analysis on the association between the number of COVID-19 cases and deaths per 100,000 inhabitants and GDP per capita and the evaluated items of the medication management of NCDs.

	Yes	Partly	No	*p*-value Kruskal-Wallis test (Wilcoxon test—pairwise comparison)
*Number of COVID-19 cases per 100,000 inhabitants in 2020, mean ± SD*				
Q8 Unlimited number of consultations with primary care is available to patients with chronic conditions without any fee	4,127.5 ± 1,653.0	NA	2,673.3 ± 1,558.1	0.017
Q9 Chronic medications are a subject of reimbursement (i.e., either patients do not pay, or pay only part of the medication cost out of their pocket)	4,135.3 ± 1,648.7	2,279.3 ± 1,232.0	NA	0.004
Q11 Face-to-face primary care and/or ambulatory specialist care appointments are limited due to COVID-19	3,655.1 ± 1,561.9	4,580.4 ± 1,818.9	1,772.9 ± 712.5	0.033 (“Yes”/“Partly” vs. “No” = 0.061; “Yes” vs. “Partly” = 0.141)
Q17 Prescriptions could be issued during teleconsultations	3,879.6 ± 1,679.7	4,693.9 ± 2,088.1	2,239.1 ± 829.5	0.048 (“Yes”/“Partly” vs. “No” = 0.048; “Yes” vs. “Partly” = 0.579)
Q25 Home delivery of prescription medication is available without visiting a pharmacy	3,869.2 ± 1,197.8	2,803 ± 1,331.1	4,591.5 ± 2,132.1	0.05 (“Yes” vs. “No” = 0.579; “Partly” vs. “No” = 0.066; “Yes” vs. “Partly” = 0.066)
Q30 Publicly available guidance for patients/repositories of information advising how to maintain chronic treatment during COVID-19, have been issued by, or approved by the national bodies, e.g., NHS	4,336.1 ± 1,512.4	4,509.6 ± 1,842.9	2,976.2 ± 1,354.6	0.034 (“Yes”/“Partly” vs. “No” = 0.053; “Yes” vs. “Partly” = 0.97)
*Number of COVID-19 deaths per 100,000 inhabitants in 2020, mean ± SD*				
Q11 Face-to-face primary care and/or ambulatory specialist care appointments are limited due to COVID-19	73.2 ± 41.9	82.0 ± 37.7	23.0 ± 17.0	0.059 (“Yes”/“Partly” vs. “No” = 0.047; “Yes” vs. “Partly” = 0.440)
Q24 Online ordering of prescription medication (i.e., medication available according to prescription only) is possible	60.9 ± 37.4	32.8 ± 37	84.3 ± 40	0.05 ^(^“Yes” vs. “No” = 0.14; “Partly” vs. “No” = 0.12; “Yes” vs. “Partly” = 0.25)
Q31 Special initiatives aimed at maintenance of chronic treatment during COVID-19 have been introduced	77.4 ± 35.0	92.5 ± 29.0	51.4 ± 38.2	0.039 (“Yes” vs. “No” = 0.102; “Partly” vs. “No” = 0.074; “Yes” vs. “Partly” = 0.343)
*GDP per capita, PPP, 2019 (current international USD), mean ± SD*				
Q12 Teleconsultations are subject of advance scheduling (e.g., you may schedule now a teleconsultation with your doctor for next Friday)	49,512.6 ± 14,060.8	47,381.7 ± 26,521.4	24,398 ± 8,908.9	0.005 (“Yes” vs. “No” <0.001; “Partly” vs. “No” = 0.036; “Yes” vs. “Partly” = 0.488)
Q17 Prescriptions could be issued during teleconsultations	49,683.9 ± 19,721.6	30,857.9 ± 16,131.1	27,830.9 ± 13,406.2	0.006 (“Yes” vs. “No” = 0.023; “Partly” vs. “No” = 1; “Yes” vs. “Partly” = 0.039)
Q18 Paper-based chronic drug prescriptions are sent to the patient by post	57,601.4 ± 14,739.2	69,992 ± 31,737.2	32,275.1 ± 11,776.5	0.001 (“Yes” vs. “No” = 0.021; “Partly” vs. “No” = 0.001; “Yes” vs. “Partly” = 0.905)
Q19 Alerting systems are available to alert the prescribers about the need to renew prescription for chronic treatment	49,620.8 ± 10,749.5	50,812.1 ± 10,866.1	38,804 ± 22,641.8	0.028 (“Yes”/“Partly” vs. “No” = 0.074; “Yes” vs. “Partly” = 0.662)
Q21 Prescriptions are possible when the patient still possesses some medication	49,239.9 ± 20,340.1	27,734 ± 12,288	47,578.2	0.005 (“Yes” vs. “No” = 0.929; “Partly” vs. “No” = 0.545; “Yes” vs. “Partly” = 0.002)
Q24 Online ordering of prescription medication (i.e., medication available according to prescription only) is possible	51,057.5 ± 14,224.5	38,338 ± 23,389.5	41,104.9 ± 25,156	0.04 (“Yes” vs. “No” = 0.03; “Partly” vs. “No” = 1; “Yes” vs. “Partly” = 0.54)
Q25 Home delivery of prescription medication is available without visiting a pharmacy	55,114.5 ± 14,812.2	37,454.6 ± 15,865.4	41,490.9 ± 28,447.3	0.011 (“Yes” vs. “No” = 0.021; “Partly” vs. “No” = 0.880; “Yes” vs. “Partly” = 0.018)

GDP: gross domestic product; NA: not applicable; PPP: purchasing power parity; SD: standard deviation.

In all countries the cost of chronic medications were fully (80%) or partly (20%) reimbursed. The mean number of COVID-19 cases per 100,000 inhabitants was significantly higher in countries with more extensive reimbursement for chronic pharmacotherapies (“Yes” response group vs. “Partly” response group *p* = 0.004) (Table 2). Electronic prescribing was available in 36 (92%) countries, and it was not found to be associated with the number of COVID-19 cases and COVID-19 deaths. Furthermore, none of the evaluated items of the patient and healthcare system regulations domain showed a significant association with GDP per capita of countries.

### Means of Communication Between the Patient and Prescriber

In 35 (90%) of countries, face-to-face primary care and/or out-patient care consultations were, at least partly, limited due to the COVID-19 pandemic. The number of COVID-19 deaths per 100,000 inhabitants showed an association with the disruption of outpatient care (“Yes” response group/“Partly” response group vs. “No” response group *p* = 0.047) (Table 2). The same trend was seen in case of the number of COVID-19 cases between response groups (*p* = 0.033); however, in pairwise comparison this association was not significant anymore (Table 2).

**FIGURE 2:**
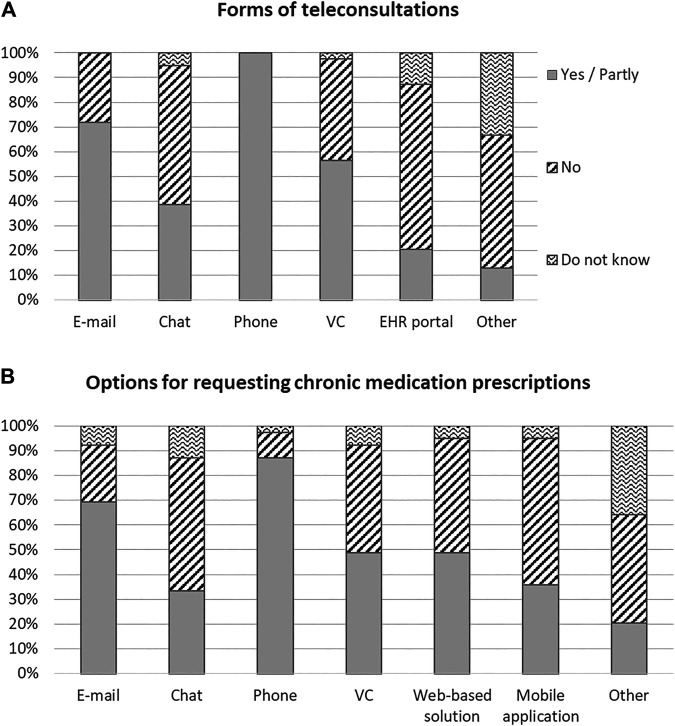
Availability of various forms of teleconsultations **(A)**, and options for requesting chronic medication prescriptions **(B)** across 39 European countries, as per December 2020. VC: videoconsultation; EHR: electronic health record.

Being able to book an advance appointment for a teleconsultation was available in 33 (85%) countries and was significantly more common in countries with higher GDP per capita (“Yes” response group vs. “No” response group *p* < 0.001, “Partly” response group vs. “No” response group *p* = 0.036) (Table 2). Across the evaluated countries the mean ± SD number of available teleconsultation services (e.g., e-mail, online chat, phone, video, and electronic health records) in the public healthcare system was 3 ± 1.3 (Figure 2A) and it was positively associated with GDP per capita of countries (*p* = 0.05). Furthermore, the mean ± SD number of teleconsultation methods for requesting prescriptions for chronic medications was 3.4 ± 1.6 (Figure 2B), but it did not show an association with any of the evaluated covariates.

### Prescriber

Access to prescribing history and/or dispensing (e.g., within electronic health record) was, at least partly, available in 33 (85%) countries and only physicians were authorized to prescribe medications in 22 (56%) countries. These items were not associated with the number of COVID-19 cases, the number of COVID-19 deaths, or country income.

Prescriptions could be issued during teleconsultations in 32 (82%) countries, significantly more common in countries with higher GDP per capita (*p* = 0.006) and in countries with higher number of COVID-19 cases (*p* = 0.048) (Table 2). Paper-based repeated drug prescriptions could be sent to the patient by post only in 9 (23%) countries. The availability of this service was associated with higher GPD per capita (“Yes” response group vs. “No” response group *p* = 0.021, “Partly” response group vs. “No” response group *p* = 0.001) (Table 2).

Systems to alert the prescribers about the need to renew prescription for chronic treatment were, at least partly, available only in 11 (28%) countries and it was found to be related to country income when comparing all response groups (*p* = 0.028) (Table 2). However, using pairwise comparison the association was not significant anymore.

### Prescription

To prescribe chronic medications for a more than a 3-months period and when the patient still has a stock of medication was possible in 26 (67%) and 37 (95%) countries, respectively. Prescribing of specialist medicines (e.g., high-cost medicines normally prescribed by specialists only) in primary care during COVID-19 pandemic was, at least partly, allowed in 12 (31%) countries. Regarding the item of medication prescription when the patient still possesses some medication, the GDP per capita of countries in the “Yes” response group was significantly higher compared to countries in the “Partly” response group (*p* = 0.002) (Table 2). No further association was found between the items of this domain and the evaluated covariates.

### Community Pharmacy Regulation

In 26 (67%) countries medication for NCDs could not be dispensed without a prescription. Online ordering and home delivery of prescription medication without visiting a pharmacy was, at least partly, available in 18 (46%) and 26 (67%) countries, respectively. Both items showed a positive association with GDP per capita (online ordering: “Yes” response group vs. “No” response group *p* = 0.03; home delivery of prescription medication: “Yes” response group vs. “No” response group *p* = 0.021). We also found weak associations between online ordering and the number of COVID-19 deaths per 100,000 inhabitants and between home delivery of prescription medication and the number of COVID-19 cases per 100,000 inhabitants when comparing all response groups; however, none of these associations remained significant in case of pairwise comparisons (Table 2).

Substitution of unavailable medication was, at least partly, allowed in 33 (85%) countries and dispensing of specialist medicines (e.g., high-cost medicines normally dispensed by hospitals) by community pharmacies during COVID-19 pandemic was made, at least partly, possible only in 8 (21%) countries. These items were not associated with the number of COVID-19 cases, the number of COVID-19 deaths or country income.

### Medication

In 35 (90%) countries no reduction of out-of-pocket costs of medication for NCDs was applied during the COVID-19 pandemic and only 21 (54%) countries applied measures specifically to address potential shortages of medicines. No significant association was found between the items of this domain and the evaluated covariates.

### Medication Taking

A national policy or specific guidance on ensuring on-going access to medication for NCDs during the COVID-19 pandemic was issued in 20 (51%) countries. In case of this variable, differences in the number of COVID-19 cases per 100,000 inhabitants between response groups were found to be significant with Kruskal-Wallis test (*p* = 0.034); however, in the pairwise comparison it was not significant anymore (Table 2).

Special initiatives for maintaining chronic pharmacotherapies were introduced in 20 (51%) countries, which was weakly associated with the number of COVID-19 deaths (*p* = 0.039), but this association did not remain significant in case of pairwise comparison (Table 2).

## Discussion

Our study offers a comprehensive “snapshot” of how different European countries responded to the challenge of assuring medication management services for NCDs during the second wave of the COVID-19 pandemic. In only half of European countries were our respondents aware of any national guidelines regarding strategies for maintaining medication availability for people with NCDs, or advice for patients on how to ensure access to medication and adherence during the pandemic. Apart from a widespread switch to remote consulting, the use of e-health solutions was variable. Electronic prescriptions were available in 92% of countries, whereas online ordering and home delivery of prescription medication were only available 46 and 67% of countries, respectively.

In line with the results of the WHO survey [World Health Organization (WHO), 2020b], and a global survey of healthcare professionals (Chudasama et al., 2020), our findings highlight that the COVID-19 pandemic limited the number of face-to-face appointments for NCDs in primary care, and out-patients. Medication management of NCDs during the COVID-19 pandemic varied between countries, which was partly explained by the differences in the structure and financing of healthcare systems across Europe. In general, greater disruption in healthcare and accelerated the uptake of e-health services was associated with greater burden of COVID-19 (Table 2). However, typically various e-health solutions were not merged into a seamless system and there may have been substantial variations even within countries. For example, in more than 80% of countries, prescriptions for NCDs could be issued without a face-to-face consultation; but home delivery of prescription medications was not available in 13 countries. In these countries, despite the availability of telemedicine services and electronic prescription, patients with NCDs could still not get their medications without leaving home. Especially in the more vulnerable patients who were shielding, it would be important to reduce the number of unnecessary personal contacts in order to reduce risk.

The COVID-19 pandemic promoted the use of e-health technologies as social distancing prevented traditional face-to-face patient-physician appointments. The available forms of teleconsultations, and options for requesting medication prescriptions were different across the evaluated countries (Figure 2). In the majority of countries, phone and e-mail were the most commonly used modes of digital communication between patients and physicians. However, in countries with higher GDP per capita, a wider range of teleconsultation (e.g., online chat, video-consultations, communication via the electronic health record portal) and e-health services (e.g., alerts when prescriptions need to be renewed, online ordering of prescription medication) were available for the management of patients with NCDs (Table 2). These results highlight the potential of innovative models of care to meet the challenges of the pandemic (Gunasekeran et al., 2021). An example of such a solution could be an alerting system for prescribers about the need to renew prescriptions for chronic treatment, reported in 11 countries. However, even simple solutions are worth introducing, in order to make the NCD patient journey easier, such as the possibility of advance scheduling of teleconsultations (missing in six countries).

Evidence suggests that the out-of-pocket cost of drugs is a significant determinant of medication adherence (Dodd et al., 2018) and a few countries reduced medication costs to offset the economic disruption of the COVID-19 pandemic. Inability to afford drugs increases the risk of non-adherence and non-persistence to chronic treatments. Allowing longer-duration prescriptions could facilitate access to drug supplies especially for patients living in remote areas, though this needs to be balanced against the risk of supply problems with “stockpiling” during the COVID-19 lockdown. Substituting unavailable medicines by a pharmacist could be a practical solution to medicine shortages. The medication supply chain is being strained by the pandemic (Shuman et al., 2020); nevertheless, only 54% of countries reported measures specifically to address potential shortages of medicines. In addition policy interventions to improve drug coverage and behavioral support consisting in patient education can improve medication adherence among people with chronic diseases (Viswanathan et al., 2012).

The reduction in the number of face-to-face appointments, and the lack or low number of interventions assuring continuous treatment of NCDs (e.g., the absence of systems to alert prescribers about the need to renew prescriptions) during the pandemic may have significant clinical consequences. This also makes economic sense as medication non-adherence leads to higher health care use and costs despite decreased drug spending (Roebuck et al., 2011). Nevertheless, half of our respondents were unaware of any national guidelines for preserving medication supplies NCDs or for patients on how to access medication and maintain adherence during the COVID-19 pandemic. These findings suggest that there is room for a system-based approach to ensure the maintenance of treatments for NCDs during crizes (such as pandemics) to minimize the consequences on long-term condition care. A European Union vision of resilient healthcare following the COVID-19 pandemic includes strengthening primary care and mental healthcare [Expert Panel on effective ways of investing in health (EXPH), 2020], bringing some hope that this ambition will become a reality. Further studies are warranted to improve understanding of the long-term clinical and economic consequences of the disruption to NCD services during the COVID-19 pandemic.

Our survey highlights major disparities in the way different European countries are dealing with the challenge of managing NCDs during the COVID-19 pandemic, and suggests strategies for improvement. Several practical and low-cost solutions (Figure 1) could be applied, as suggested in our quick commentary (Kardas et al., 2021), including: 1) an increased range of remote services for ordering repeat prescriptions (e.g., online, via mobile app), 2) expanding the scope of professionals authorized to prescribe (or issue repeat prescriptions), 3) increasing the duration of prescriptions (though this needs to be balanced with managing shortages), 4) enabling community pharmacies to dispense medications normally restricted to hospitals, 5) allowing substitution of unavailable drugs, 6) creating e-health systems supporting patients in long-term treatment, encouraging patient empowerment and patient-centred care, and 7) providing publicly available guidance on strategies for maintaining treatment during pandemic lockdown. Our findings point to interventions worth taking during COVID-19 pandemic in order to maintain NCDs treatment. It might be hypothesized; however, that these activities are also important beyond the current pandemic, as healthcare systems adapt to an aging population with increasing multimorbidity [Chatterji et al., 2015; World Health Organization (WHO), 2018b].

Our results should be considered in light of the following limitations. The self-developed questionnaire and multiple-choice questions with closed answers allowed us to seek very specific information; however, it may be biased by the authors’ preconceived perceptions. To minimize this risk, external experts were asked to assess the questionnaire with respect to the content, construct and criterion validity. The survey was completed by members of ENABLE who were asked to provide information on the current practices in their own countries rather than regional, local or their own perspective. In 12 countries we had more than one respondent; however, in some countries information was based on the answers of one respondent, which might lead to bias. The majority of participants were working at the academic sector of medical or pharmaceutical sciences with more than 20 years of work experience, and their views represents one perspective while there may be substantial differences in primary healthcare and medicines even between regions within a country. It should also be noted that the regulations and reimbursement of primary care and pharmacy practice are changing over time, and especially digital solutions were rapidly introduced in healthcare in many countries already before the COVID-19 outbreak. Consequently, this survey represents the situation at a single point of time and the replies for some questions may have differed substantially if the survey had been distributed at another time point.

To conclude, our survey has identified marked disparities in ensuring medication management services for NCDs across Europe. This points to the need for a paradigm shift in NCD-related healthcare services to assure access and enable adherence to chronic pharmacotherapies during the current pandemic (and future disasters). E-health solutions cannot solve all the challenges of NCD care (e.g., some therapies need regular blood tests) and safe arrangements will be needed. In the short-term, we must optimize the health of NCD patients at risk of poor outcomes from COVID-19. In the long-term, maintaining access to NCD treatments could limit the negative consequences of disruptive health service events.

## ENABLE Collaborators Participating in the Study Were

Darinka Gjorgieva Ackova, Tamás Ágh, Adriana Baban, Martina Bago, Juris Barzdins, Noemi Bitterman, Gregor Bond, Job FM van Boven, Yasemin Çayır, Ioanna Chouvarda, Maria Cordina, Alexandru Corlateanu, Jaime Correia de Sousa, Petra Denig, Dragana Drakul, Natasa Duborija-Kovacevic, Çi̇ğdem GamzeÖzkan, Cristina Ghiciuc, Catherine Goetzinger, Anne Gerd Granas, Joao Gregorio, Jolanta Gulbinovic, Maja Ortner Hadžiabdić, Freyja Jónsdóttir, Przemyslaw Kardas, Maria Kamusheva, Elena Kkolou, Mitja Kos, Ott Laius, Fedor Lehocki, Francisca Leiva, Urska Nabergoj Makovec, Katerina Mala-Ladova, Enrica Menditto, Vildan Mevsim, Jovan Mihajlovic, Valentina Orlando, Christos Petrou, Guenka Petrova, Hilary Pinnock, Mitar Popović, Richard Reilly, Susanne Reventlow, Marie Schneider, Ivana Tadic, Ugo Trama, Indre Treciokiene, Ioanna Tsiligianni, Esra Uslu, Eric van Ganse, Jiří Vlček, Daisy Volmer, Vesna Vujic-Aleksic, Björn Wettermark.

## Data Availability

The raw data supporting the conclusions of this article will be made available by the authors, without undue reservation.
