# Supplementary material for: A Cross-Sectional Survey on Medication Management Practices for Noncommunicable Diseases in Europe During the Second Wave of the COVID-19 Pandemic
Source: Front Pharmacol. 2021 Jun 7;12:685696. doi: 10.3389/fphar.2021.685696 (PMC8216671; doi:10.3389/fphar.2021.685696)
Supplement: Supplementary file 1 [file DataSheet1.PDF]

A cross-sectional survey on medication management practices for noncommunicable diseases in Europe during the second wave of the COVID-19 pandemic

Supplementary material

Survey Questionnaire

Figure S1. Survey Questionnaire

cost ENABLE

TherapyMaintenance@COVID

Your data

* 1. Your data

Name

Organisation

Country

Email Address

* 2. Your Gender

- Male
- Female
- Prefer not to answer

* 3. Your overall work experience (years)

- 0-9
- 10-19
- 20-29
- 30+
- Prefer not to answer

* 4. What is your primary field of work?

- Clinical / Healthcare
- Academia (research or education)
- Health Insurance / Regulatory Agency
- Government / Health Administration / Health Authority
- Commercial company / industry
- Other (please specify)

Other (please specify)

cost ENABLE

TherapyMaintenance@COVID

1. Patient & healthcare system regulations in YOUR COUNTRY

Answering questions provided below, please take NATIONAL perspective, i.e. the scenario that is typical for your entire country.

* 5. Public healthcare system in your country is available to all citizens

- Yes
- No
- I do not know

* 6. Public healthcare system in your country is covering ambulatory care

- Yes
- Partly
- No
- I do not know

* 7. Primary healthcare is included in the public healthcare system in your country

- Yes
- Partly
- No
- I do not know

* 8. Unlimited number of consultations with primary care is available to patients with chronic conditions, without any fee

- Yes
- No
- I do not know

* 9. Chronic medications are a subject of reimbursement (i.e. either patients do not pay, or pay only part of the medication cost out of their pocket)

- Yes
- Partly
- No
- I do not know

* 10. Electronic prescriptions are available

- Yes
- Partly
- No
- I do not know

cost ENABLE

TherapyMaintenance@COVID

2. Means of communication

* 11. Face-to-face primary care and/or ambulatory specialist care appointments are limited due to COVID-19

- Yes (please specify below)
- Partly (please specify below)
- No
- I do not know

please specify

* 12. Teleconsultations are subject of advance scheduling (e.g. you may schedule now a teleconsultation with your doctor for next Friday)

- Yes
- Partly
- No
- I do not know

* 13. Which, out of teleconsultation options provided below, are available and accepted as a regular means of patient-doctor contact in the public healthcare system?

	Yes	Partly	No	I do not know
e-mail				
online chat				
phone				
video				
through an EHR portal *(EHR - electronic health record)*				
other (please specify)				

please specify here

* 14. Which option of requesting the prescriptions for chronic medication are available?

	Yes	Partly	No	I do not know
over e-mail	◯	◯	◯	◯
over chat	◯	◯	◯	◯
over phone	◯	◯	◯	◯
over videoconsultation	◯	◯	◯	◯
over web-based solution	◯	◯	◯	◯
over dedicated mobile application	◯	◯	◯	◯
over other means (please specify)	◯	◯	◯	◯

please specify here

cost ENABLE

TherapyMaintenance@COVID

3. Prescriber

* 15. Access to history of medication (prescribing and/or dispensing) is available to the other prescribers (e.g. within electronic health record)

- ◯ Yes (please specify below)
- ◯ Partly
- ◯ No
- ◯ I do not know

please specify

[ ]

* 16. Prescribers other than physicians are authorised to prescribe

- ◯ Yes (please specify below)
- ◯ Partly
- ◯ No
- ◯ I do not know

Who? please specify

[ ]

* 17. Prescriptions could be issued during teleconsultations

- ◯ Yes
- ◯ Partly
- ◯ No
- ◯ I do not know

* 18. Paper-based chronic drug prescriptions are sent to the patient by post

- ◯ Yes
- ◯ Partly
- ◯ No
- ◯ Not applicable (paper-based prescriptions are no more available)
- ◯ I do not know

* 19. Alerting systems are available to alert the prescribers about the need to renew prescription for chronic treatment

- Yes
- Partly
- No
- I do not know

ENABLE

TherapyMaintenance@COVID

4. Prescription

* 20. Prescriptions for longer periods (≥ 3 months) are possible at a time

- Yes
- Partly
- No
- I do not know

* 21. Prescriptions are possible when the patient still possesses some medication

- Yes
- Partly
- No
- I do not know

* 22. Prescribing of specialist medicines (e.g. high-cost medicines normally prescribed by dedicated specialists only) in primary care during COVID-19 pandemic has been made possible

- Yes (please specify below)
- Partly (please specify below)
- No
- I do not know

please specify

cost ENABLE

TherapyMaintenance@COVID

5. Pharmacy

* 23. Chronic medication dispensing without prescription is available (with reimbursement applied)

- Yes
- Partly
- No
- I do not know

* 24. Online ordering of prescription medication (i.e. medication available according to prescription only) is possible

- Yes
- Partly
- No
- I do not know

* 25. Home delivery of prescription medication is available without visiting a pharmacy

- Yes
- Partly
- No
- I do not know

* 26. Substitution of unavailable medicines with equivalent one is allowed without a need for a new prescription

- Yes
- Partly
- No
- I do not know

* 27. Dispensing of specialist medicines (e.g. high-cost medicines normally dispensed by hospitals) by community pharmacies during COVID-19 pandemic has been made possible

- Yes (please specify below)
- Partly (please specify below)
- No
- I do not know

please specify

TherapyMaintenance@COVID

6. Medication

* 28. Special reduction of out-of-pocket costs of chronic medication during COVID-19 pandemic has been applied (e.g. due to higher reimbursement, etc.)

- Yes (please specify below)
- Partly (please specify below)
- No
- I do not know

please specify

* 29. Measures to address possible shortages of medicines during the COVID-19 pandemic have been applied

- Yes (please specify below)
- Partly (please specify below)
- No
- I do not know

please specify

TherapyMaintenance@COVID

7. Medication taking

* 30. Publicly available guidance for patients / repositories of information advising how to maintain chronic treatment during COVID-19, have been issued by, or approved by the national bodies, e.g. NHS

- ◯ Yes (please specify below)
- ◯ Partly
- ◯ No
- ◯ I do not know

Other (please specify)

* 31. Special initiatives aimed at maintenance of chronic treatment during COVID-19 have been introduced (give example)

- ◯ Yes
- ◯ Partly
- ◯ No
- ◯ I do not know

please specify

Country-specific responses for each item of the survey questionnaire

Figure S2. Country specific responses for items of “The patient and healthcare regulations” domain

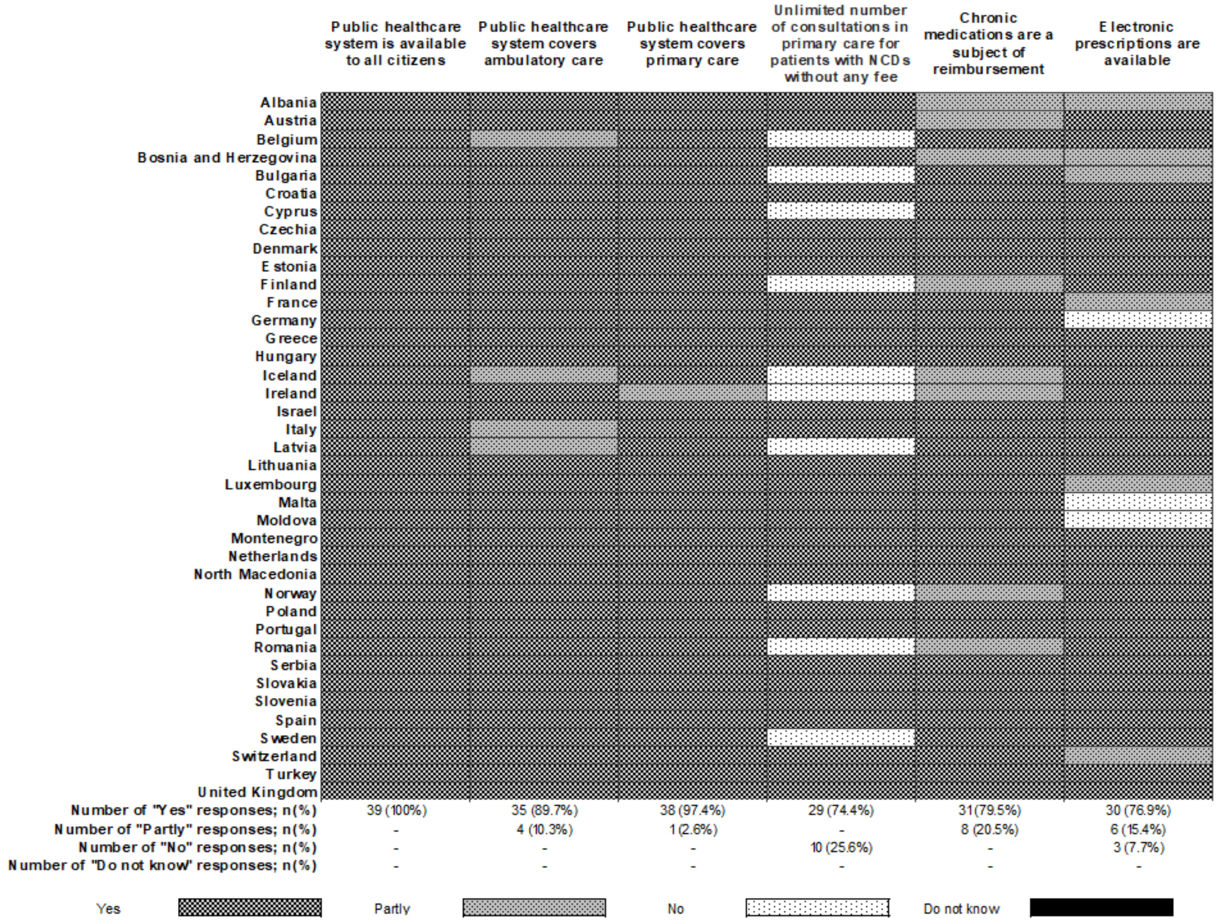

NCD: non-communicable disease

Figure S3. Country specific responses for items of "Means of communication between the patient and prescriber" domain

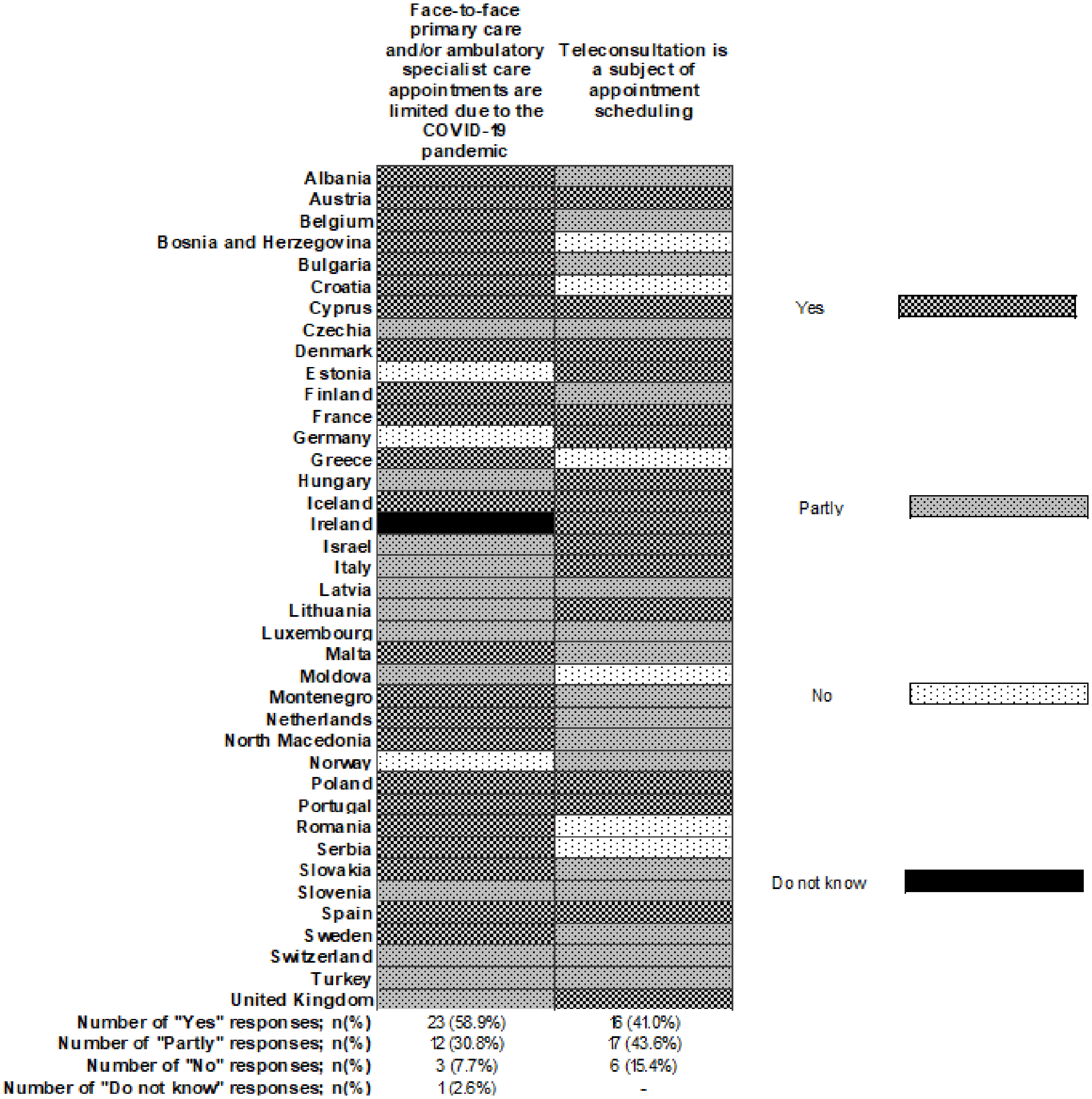

Figure S4. Availability of various forms of teleconsultations (A), and options for requesting chronic medication prescriptions (B)

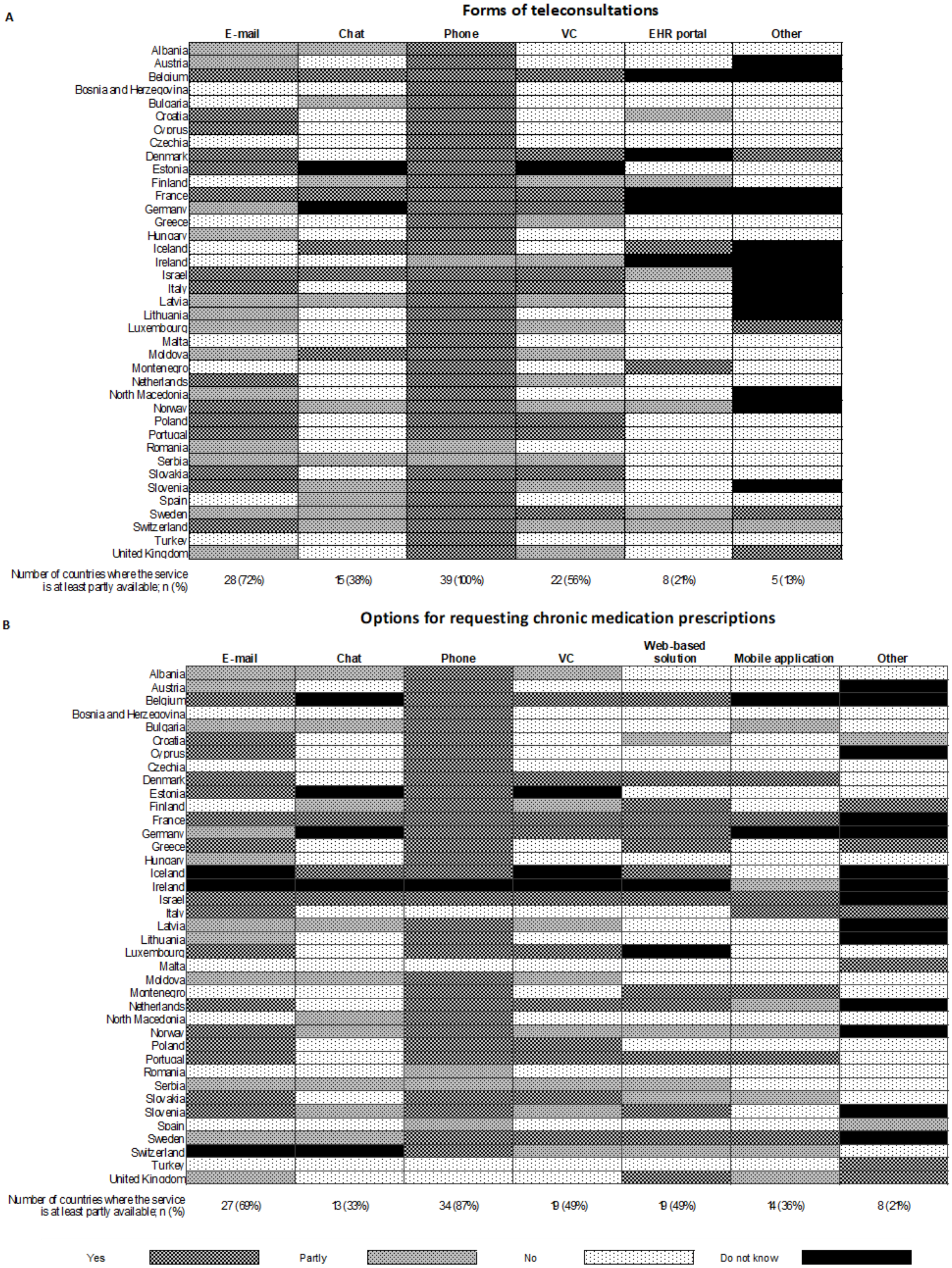

VC: videoconsultation; EHR: electronic health record

Figure S5. Country specific responses for items of “Prescriber” domain

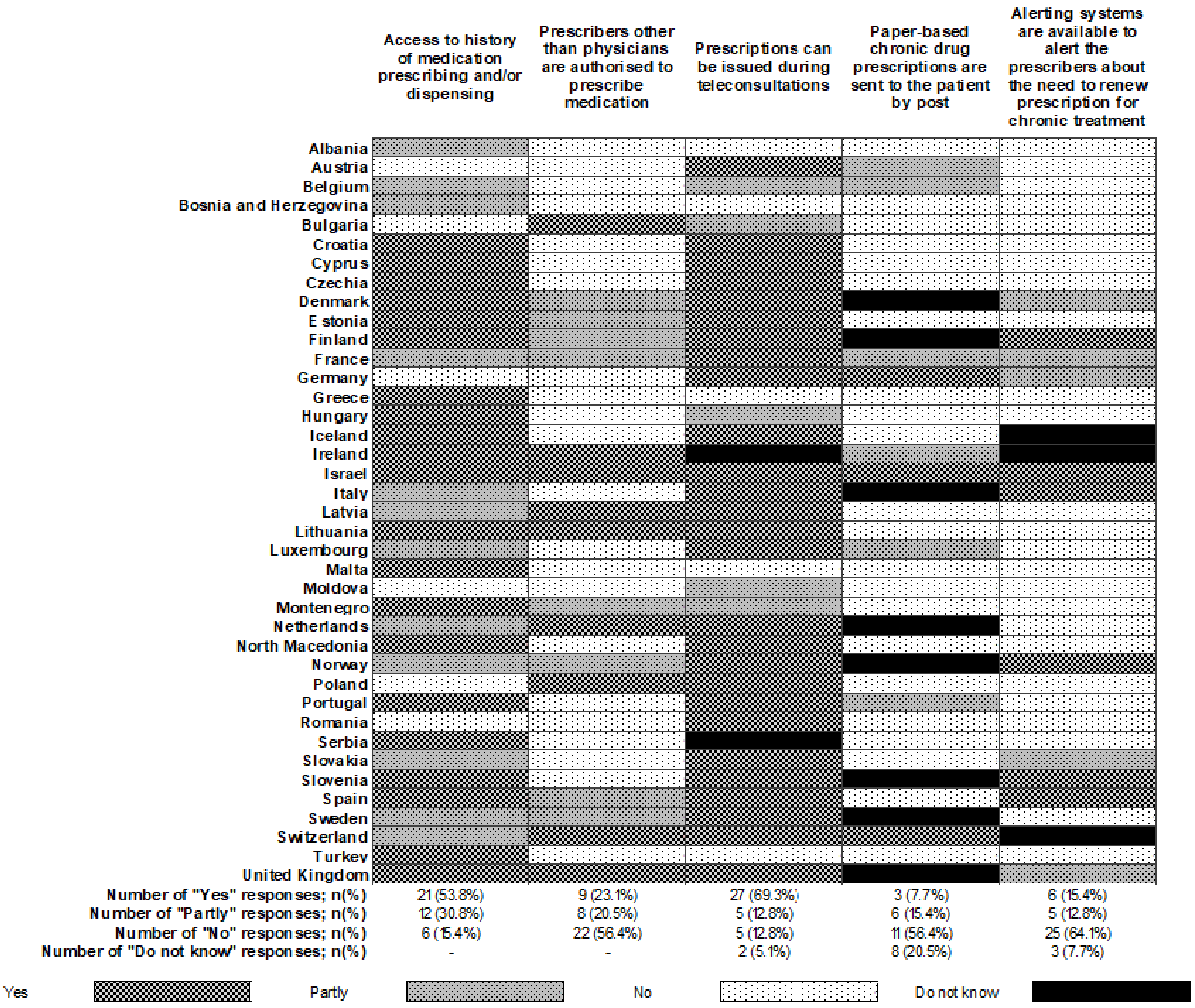

Figure S6. Country specific responses for items of "Prescription" domain

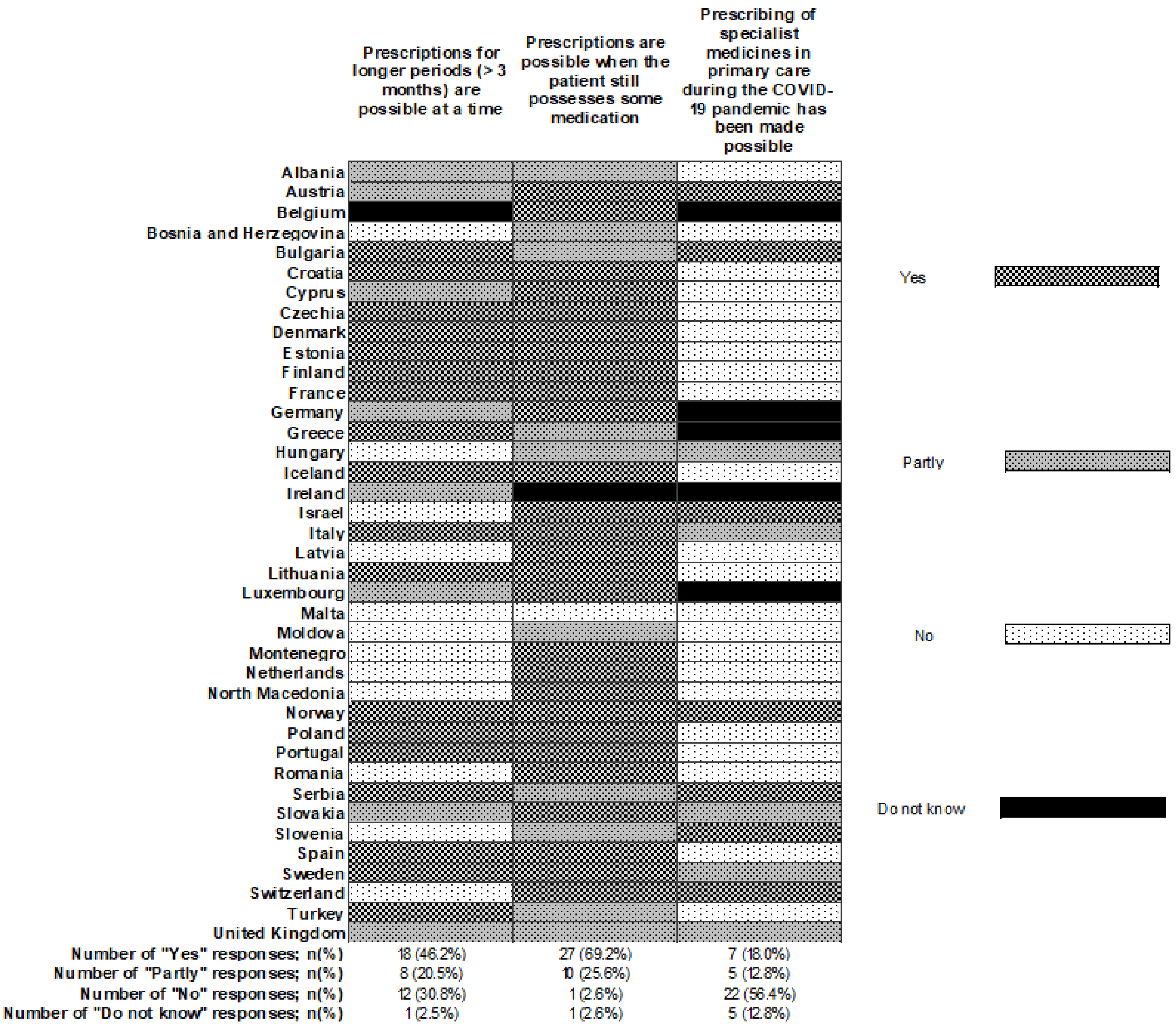

Figure S7. Country specific responses for items of "Community pharmacy regulations" domain

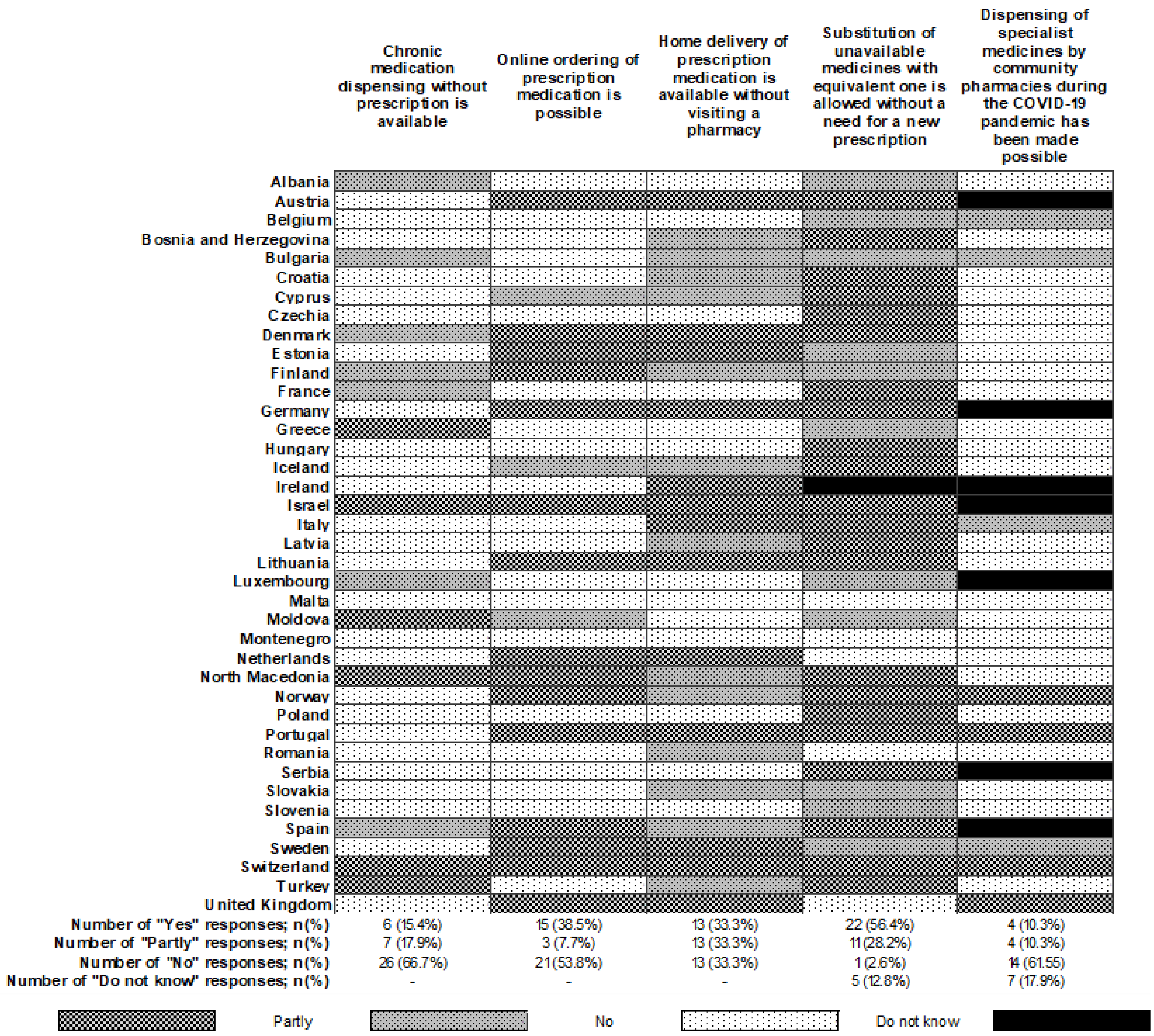

Figure S8. Country specific responses for items of “Medication” domain

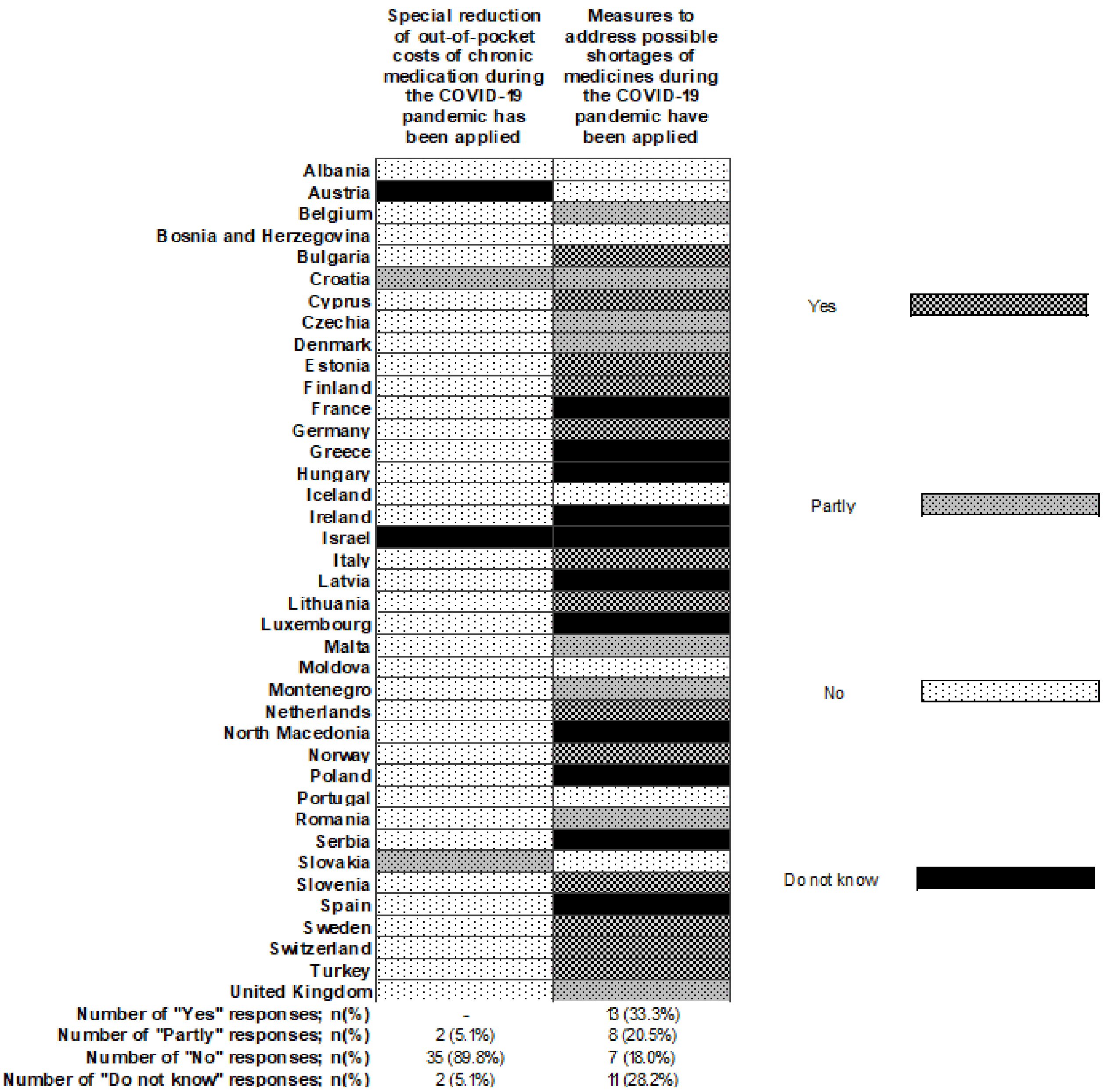

Figure S9. Country specific responses for items of “Medication taking” domain

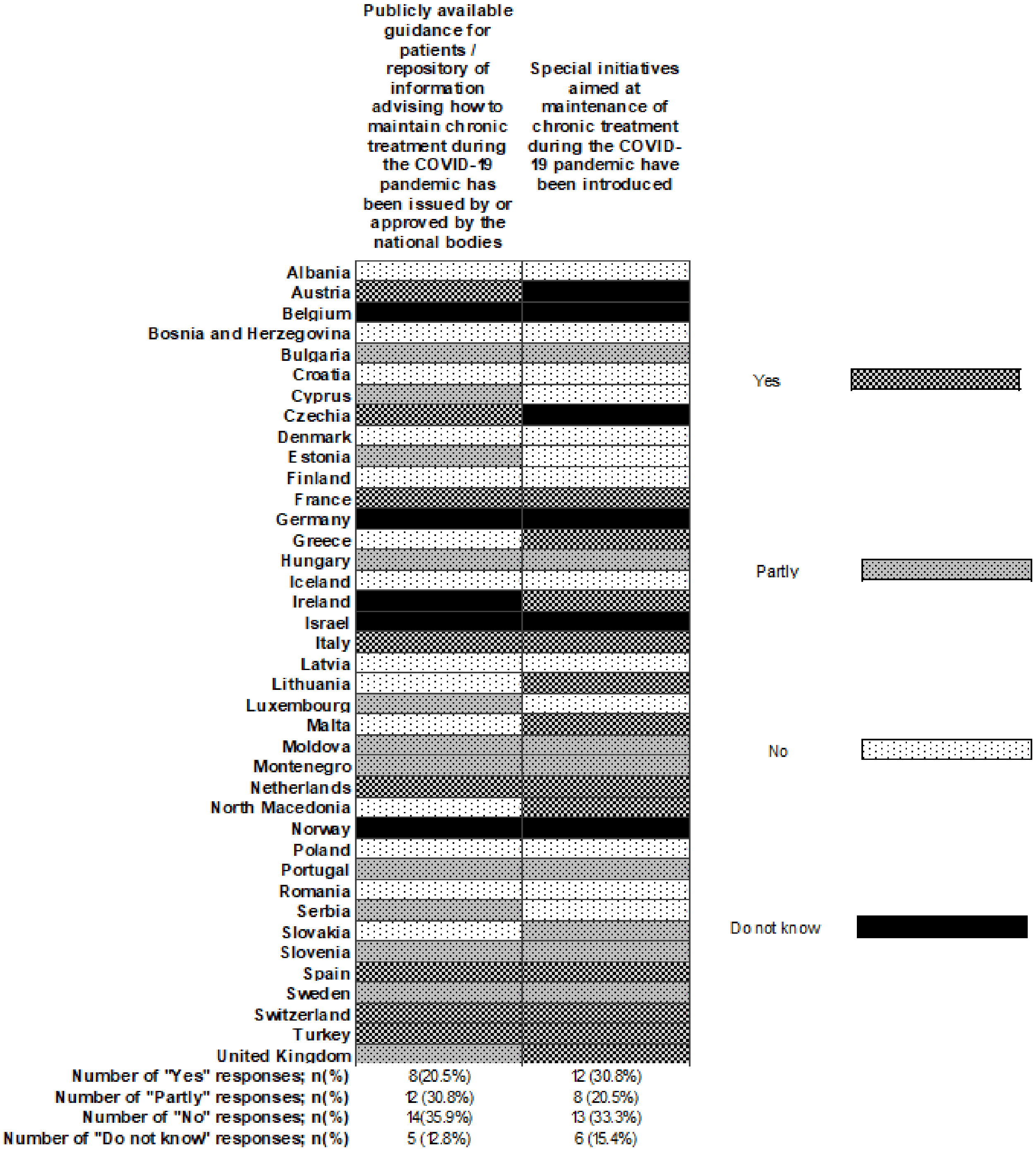

Detailed results of the statistical analysis

Table S1. Results of the statistical analysis related to items of patient & healthcare system regulations domain

	Yes	Partly	No	P-value
**Q5 Public healthcare system in your country is available to all citizens**				
N of COVID-19 cases per 100,000 inhabitants in 2020, mean ± SD	3754.6 ± 1732.6	NA	NA	NA
N of COVID-19 deaths per 100,000 inhabitants in 2020, mean ± SD	71.4 ± 41.0	NA	NA	NA
GDP per capita, PPP, 2019 (current international $), mean ± SD	44720 ± 21510.4	NA	NA	NA
**Q6 Public healthcare system in your country is covering ambulatory care**				
N of COVID-19 cases per 100,000 inhabitants in 2020, mean ± SD	3810.0 ± 1742.7	3269.3 ± 1804.6	NA	0.579
N of COVID-19 deaths per 100,000 inhabitants in 2020, mean ± SD	69.8 ± 36.6	85.2 ± 76.6	NA	0.853
GDP per capita, PPP, 2019 (current international $), mean ± SD	44253 ± 22402.8	48806.1 ± 12163.6	NA	0.459
**Q7 Primary healthcare is included in the public healthcare system in your country**				
N of COVID-19 cases per 100,000 inhabitants in 2020, mean ± SD	3798.7 ± 1733.5	2077.5	NA	0.248
N of COVID-19 deaths per 100,000 inhabitants in 2020, mean ± SD	72.0 ± 41.4	46.1	NA	0.534

GDP per capita, PPP, 2019 (current international $), mean ± SD	43536.7 ± 20472.5	89683.6	NA	0.110
**Q8 Unlimited number of consultations with primary care is available to patients with chronic conditions without any fee**				
N of COVID-19 cases per 100,000 inhabitants in 2020, mean ± SD	4127.5 ± 1653.0	NA	2673.3 ± 1558.1	**0.017**
N of COVID-19 deaths per 100,000 inhabitants in 2020, mean ± SD	76.1 ± 35.1	NA	57.5 ± 54.7	0.108
GDP per capita, PPP, 2019 (current international $), mean ± SD	42247.5 ± 21960.1	NA	51890.1 ± 19402.6	0.177
**Q9 Chronic medications are a subject of reimbursement (i.e. either patients do not pay, or pay only part of the medication cost out of their pocket)**				
N of COVID-19 cases per 100,000 inhabitants in 2020, mean ± SD	4135.3 ± 1648.7	2279.3 ± 1232.0	NA	**0.004**
N of COVID-19 deaths per 100,000 inhabitants in 2020, mean ± SD	77.1 ± 39.4	49.2 ± 42.0	NA	0.082
GDP per capita, PPP, 2019 (current international $), mean ± SD	43431.9 ± 20389.83	49711.07 ± 26347.58	NA	0.404
**Q10 Electronic prescriptions are available**				
N of COVID-19 cases per 100,000 inhabitants in 2020, mean ± SD	3754.3 ± 1749.7	4232.7 ± 2004.0	2801.8 ± 750.2	0.555
N of COVID-19 deaths per 100,000 inhabitants in 2020, mean ± SD	69.3 ± 43.9	90.2 ± 28.8	53.8 ± 18.6	0.302

GDP per capita, PPP, 2019 (current international $), mean ± SD	44037.8 ± 15709.2	50701.5 ± 42563.2	39578.5 ± 23018.9	0.965

Table S2. Results of the statistical analysis related to items of means of communication between the patient and prescriber domain

	**Yes**	**Partly**	**No**	**P-value**
**Q11 Face-to-face primary care and/or ambulatory specialist care appointments are limited due to COVID-19**				
N of COVID-19 cases per 100,000 inhabitants in 2020, mean ± SD	3655.1 ± 1561.9	4580.4 ± 1818.9	1772.9 ± 712.5	**0.033**[a]
N of COVID-19 deaths per 100,000 inhabitants in 2020, mean ± SD	73.2 ± 41.9	82.0 ± 37.7	23.0 ± 17.0	0.059[b]
GDP per capita, PPP, 2019 (current international $), mean ± SD	39776.7 ± 15805.2	47667.4 ± 27916.8	55840.8 ± 15081	0.340
**Q12 Teleconsultations are subject of advance scheduling (e.g. you may schedule now a teleconsultation with your doctor for next Friday)**				
N of COVID-19 cases per 100,000 inhabitants in 2020, mean ± SD	3432.3 ± 1079.3	4099.7 ± 2278.1	3636.2 ± 1400.0	0.763
N of COVID-19 deaths per 100,000 inhabitants in 2020, mean ± SD	62.7 ± 38.0	76.6 ± 47.1	79.6 ± 30.7	0.503
GDP per capita, PPP, 2019 (current international $), mean ± SD	49512.6 ± 14060.8	47381.7 ± 26521.4	24398 ± 8908.9	**0.005**[c]

[a] P-value “Yes” / “Partly” vs “No” = 0.061; P-value “Yes” vs. “Partly” = 0.141

[b] P-value “Yes” / “Partly” vs “No” = **0.047**; P-value “Yes” vs. “Partly” = 0.440

[c] P-value “Yes” vs “No” **<0.001**; P-value “Partly” vs “No” = **0.036**; P-value “Yes” vs. “Partly” = 0.488

Q13 Which, out of teleconsultation options provided below, are available and accepted as a regular means of patient-doctor contact in the public healthcare system? ("Yes"/"Partly" vs. "No")				
**Covariates**	**Estimate**	**95% CI**		**P-value**
N of COVID-19 cases per 100,000 inhabitants in 2020, mean ± SD	0.00003	-0.00007	0.0001	0.544
N of COVID-19 deaths per 100,000 inhabitants in 2020, mean ± SD	-0.0009	-0.0047	0.0028	0.623
GDP per capita, PPP, 2019 (current international $), mean ± SD	0.000006	-0.0000002	0.000001	**0.05**

Q14 Which option of requesting the prescriptions for chronic medication are available? ("Yes"/"Partly" vs. "No")				
**Covariates**	**Estimate**	**95% CI**		**P-value**
N of COVID-19 cases per 100,000 inhabitants in 2020, mean ± SD	0.00003	-0.00008	0.0001	0.565
N of COVID-19 deaths per 100,000 inhabitants in 2020, mean ± SD	-0.0012	-0.0005	0.0027	0.394
GDP per capita, PPP, 2019 (current international $), mean ± SD	0.000001	-0.000006	0.000008	0.774

Table S3. Results of the statistical analysis related to items of prescriber domain

	Yes	Partly	No	P-value
**Q15 Access to history of medication (prescribing and/or dispensing) is available to the other prescribers (e.g. within electronic health record)**				
N of COVID-19 cases per 100,000 inhabitants in 2020, mean ± SD	3770.2 ± 1921.3	3975.1 ± 1806.3	3258.9 ± 677.7	0.715
N of COVID-19 deaths per 100,000 inhabitants in 2020, mean ± SD	64.4 ± 42.6	81.2 ± 45.6	76.1 ± 21.8	0.602
GDP per capita, PPP, 2019 (current international $), mean ± SD	42059.9 ± 15982.2	52952.3 ± 29445.2	37565.4 ± 18262.8	0.354
**Q16 Prescribers other than physicians are authorised to prescribe**				
N of COVID-19 cases per 100,000 inhabitants in 2020, mean ± SD	3910 ± 1306.7	3423.7 ± 2321.1	3811.3 ± 1711.9	0.758
N of COVID-19 deaths per 100,000 inhabitants in 2020, mean ± SD	69 ± 29.1	58.2 ± 46.6	77.1 ± 43.5	0.548
GDP per capita, PPP, 2019 (current international $), mean ± SD	49965.4 ± 20829.0	50051.3 ± 14233.4	40635.5 ± 23756.2	0.145
**Q17 Prescriptions could be issued during teleconsultations**				
N of COVID-19 cases per 100,000 inhabitants in 2020, mean ± SD	3879.6 ± 1679.7	4693.9 ± 2088.1	2239.1 ± 829.5	**0.048**[a]
N of COVID-19 deaths per 100,000 inhabitants in 2020, mean ± SD	67.9 ± 39.7	114 ± 35.6	56.9 ± 39.4	0.103
GDP per capita, PPP, 2019 (current international $), mean ± SD	49683.9 ± 19721.6	30857.9 ± 16131.1	27830.9 ± 13406.2	**0.006**[b]

**Q18 Paper-based chronic drug prescriptions are sent to the patient by post**				
N of COVID-19 cases per 100,000 inhabitants in 2020, mean ± SD	4209 ± 1798.1	4606.2 ± 1877.6	3577.2 ± 1678.9	0.307
N of COVID-19 deaths per 100,000 inhabitants in 2020, mean ± SD	55.3 ± 25.5	89.6 ± 43.9	68.4 ± 38.2	0.484
GDP per capita, PPP, 2019 (current international $), mean ± SD	57601.4 ± 14739.2	69992 ± 31737.2	32275.1 ± 11776.5	**0.001**[c]
**Q19 Alerting systems are available to alert the prescribers about the need to renew prescription for chronic treatment**				
N of COVID-19 cases per 100,000 inhabitants in 2020, mean ± SD	3420.4 ± 2191.3	3288.4 ± 779.9	4015.8 ± 1757.5	0.699
N of COVID-19 deaths per 100,000 inhabitants in 2020, mean ± SD	71.8 ± 59.2	64.2 ± 38.4	75.7 ± 38.2	0.829
GDP per capita, PPP, 2019 (current international $), mean ± SD	49620.8 ± 10749.5	50812.1 ± 10866.1	38804 ± 22641.8	**0.028**[d]

[a] P-value “Yes” vs “No” = **0.048**; P-value “Partly” vs “No” = **0.048**; P-value “Yes” vs. “Partly” = 0.579

[b] P-value “Yes” vs “No” = **0.023**; P-value “Partly” vs “No” = 1; P-value “Yes” vs. “Partly” = **0.039**

[c] P-value “Yes” vs “No” = **0.021**; P-value “Partly” vs “No” = **0.001**; P-value “Yes” vs. “Partly” = 0.905

[d] P-value “Yes” / “Partly” vs “No” = 0.074; P-value “Yes” vs. “Partly” = 0.662

Table S4. Results of the statistical analysis related to items of prescription domain

	Yes	Partly	No	P-value
**Q20 Prescriptions for longer periods (> 3 months) are possible at a time**				
N of COVID-19 cases per 100,000 inhabitants in 2020, mean ± SD	3373 ± 1731.3	3527.8 ± 1861.5	4317.6 ± 1604.4	0.344
N of COVID-19 deaths per 100,000 inhabitants in 2020, mean ± SD	62.6 ± 40.8	57 ± 30.2	85.7 ± 35	0.2337
GDP per capita, PPP, 2019 (current international $), mean ± SD	43122.4 ± 13702.9	58946.6 ± 34354	36662.9 ± 17963.7	0.194
**Q21 Prescriptions are possible when the patient still possesses some medication**				
N of COVID-19 cases per 100,000 inhabitants in 2020, mean ± SD	4011.3 ± 1835.9	3339.6 ± 1449.5	2650.5	0.354
N of COVID-19 deaths per 100,000 inhabitants in 2020, mean ± SD	69.1 ± 42.3	82.6 ± 40.2	44.6	0.449
GDP per capita, PPP, 2019 (current international $), mean ± SD	49239.9 ± 20340.1	27734 ± 12288	47578.2	**0.005**[a]
**Q22 Prescribing of specialist medicines (e.g. high-cost medicines normally prescribed by dedicated specialists only) in primary care during COVID-19 pandemic has been made possible**				
N of COVID-19 cases per 100,000 inhabitants in 2020, mean ± SD	4206.1 ± 1752.3	3792.4 ± 505.9	3599 ± 1724	0.419
N of COVID-19 deaths per 100,000 inhabitants in 2020, mean ± SD	71.4 ± 44.2	94.7 ± 30.3	64.6 ± 39.2	0.269
GDP per capita, PPP, 2019 (current international $), mean ± SD	47562.3 ± 20879.1	44153.7 ± 9863.2	37714.3 ± 14784.6	0.3676

[a] P-value “Yes” vs “No” = 0.929; P-value “Partly” vs “No” = 0.545; P-value “Yes” vs. “Partly” = 0.002

Table S5. Results of the statistical analysis related to items of community pharmacy regulations domain

	Yes	Partly	No	P-value
**Q23 Chronic medication dispensing without prescription is available (with reimbursement applied)**				
N of COVID-19 cases per 100,000 inhabitants in 2020, mean ± SD	3530.8 ± 1695.6	3475.9 ± 2182.5	3881.3 ± 1671.7	0.767
N of COVID-19 deaths per 100,000 inhabitants in 2020, mean ± SD	65.6 ± 35.3	67.7 ± 41.7	73.7 ± 43.3	0.878
GDP per capita, PPP, 2019 (current international $), mean ± SD	34608 ± 21231.8	53471.6 ± 35445.1	44697.3 ± 16298.1	0.287
**Q24 Online ordering of prescription medication (i.e. medication available according to prescription only) is possible**				
N of COVID-19 cases per 100,000 inhabitants in 2020, mean ± SD	3632.7 ± 1510.8	2655 ± 1004.7	3998.7 ± 1936.5	0.474
N of COVID-19 deaths per 100,000 inhabitants in 2020, mean ± SD	60.9 ± 37.4	32.8 ± 37	84.3 ± 40	**0.05**[a]
GDP per capita, PPP, 2019 (current international $), mean ± SD	51057.5 ± 14224.5	38338 ± 23389.5	41104.9 ± 25156	**0.04**[b]
**Q25 Home delivery of prescription medication is available without visiting a pharmacy**				
N of COVID-19 cases per 100,000 inhabitants in 2020, mean ± SD	3869.2 ± 1197.8	2803 ± 1331.1	4591.5 ± 2132.1	**0.05**[c]
N of COVID-19 deaths per 100,000 inhabitants in 2020, mean ± SD	64.3 ± 32.2	61.4 ± 47.3	88.4 ± 39.7	0.208
GDP per capita, PPP, 2019 (current international $), mean ± SD	55114.5 ± 14812.2	37454.6 ± 15865.4	41490.9 ± 28447.3	**0.011**[d]
**Q26 Substitution of unavailable medicines with equivalent one is allowed without a need for a new prescription**				

N of COVID-19 cases per 100,000 inhabitants in 2020, mean ± SD	3707.0 ± 1467.4	3655.6 ± 2149.1	4517.1 ± 2049.2	0.789
N of COVID-19 deaths per 100,000 inhabitants in 2020, mean ± SD	67.7 ± 40.0	75.4 ± 50.1	83.5 ± 29.0	0.734
GDP per capita, PPP, 2019 (current international $), mean ± SD	42975.2 ± 15959.4	44797.3 ± 30459.6	43234.1 ± 14629	0.818
**Q27 Dispensing of specialist medicines (e.g. high-cost medicines normally dispensed by hospitals) by community pharmacies during COVID-19 pandemic has been made possible**				
N of COVID-19 cases per 100,000 inhabitants in 2020, mean ± SD	3618.1 ± 1883	4186.1 ± 1215.2	3542.4 ± 1781.9	0.5538
N of COVID-19 deaths per 100,000 inhabitants in 2020, mean ± SD	68.7 ± 44.0	123.7 ± 35.9	65.7 ± 40.6	0.084
GDP per capita, PPP, 2019 (current international $), mean ± SD	57558 ± 16516.7	46003.8 ± 14699.2	37155 ± 14178.7	0.075

[a] P-value "Yes" vs "No" = 0.14; P-value "Partly" vs "No" = 0.12; P-value "Yes" vs. "Partly" = 0.25

[b] P-value "Yes" vs "No" = **0.03**; P-value "Partly" vs "No" = 1; P-value "Yes" vs. "Partly" = 0.54

[c] P-value "Yes" vs "No" = 0.579; P-value "Partly" vs "No" = 0.066; P-value "Yes" vs. "Partly" = 0.066

[d] P-value "Yes" vs "No" = **0.021**; P-value "Partly" vs "No" = 0.880; P-value "Yes" vs. "Partly" = **0.018**

Table S6. Results of the statistical analysis related to items of medication domain

	Yes	Partly	No	P-value
**Q28 Special reduction of out-of-pocket costs of chronic medication during COVID-19 pandemic has been applied (e.g. due to higher reimbursement, etc.)**				
N of COVID-19 cases per 100,000 inhabitants in 2020, mean ± SD	NA	4337.7 ± 1253.9	3672.3 ± 1795.6	0.502

N of COVID-19 deaths per 100,000 inhabitants in 2020, mean ± SD	NA	73.1 ± 37.9	72.2 ± 42.5	0.893
GDP per capita, PPP, 2019 (current international $), mean ± SD	NA	32323.4 ± 1686.5	45031.9 ± 22373.2	0.283
**Q29 Measures to address possible shortages of medicines during the COVID-19 pandemic have been applied**				
N of COVID-19 cases per 100,000 inhabitants in 2020, mean ± SD	3306.7 ± 1769.5	4832.8 ± 1950.1	3208.8 ± 985.7	0.223
N of COVID-19 deaths per 100,000 inhabitants in 2020, mean ± SD	60.2 ± 45.5	94.9 ± 45.8	62.4 ± 45.8	0.222
GDP per capita, PPP, 2019 (current international $), mean ± SD	48758.2 ± 14602.1	43593.8 ± 13116.5	33792.7 ± 20409.7	0.235

Table S7. Results of the statistical analysis related to items of medication taking domain

	Yes	Partly	No	P-value
**Q30 Publicly available guidance for patients / repositories of information advising how to maintain chronic treatment during COVID-19, have been issued by, or approved by the national bodies, e.g. NHS**				
N of COVID-19 cases per 100,000 inhabitants in 2020, mean ± SD	4336.1 ± 1512.4	4509.6 ± 1842.9	2976.2 ± 1354.6	**0.034**[a]
N of COVID-19 deaths per 100,000 inhabitants in 2020, mean ± SD	86.5 ± 31.8	80.9 ± 38.2	58.0 ± 37.9	0.161
GDP per capita, PPP, 2019 (current international $), mean ± SD	50839.9 ± 13644.5	42515.01 ± 28728.6	36479.5 ± 15008.2	0.087
**Q31 Special initiatives aimed at maintenance of chronic treatment during COVID-19 have been introduced (give example)**				
N of COVID-19 cases per 100,000 inhabitants in 2020, mean ± SD	3576.8 ± 1353.1	4504.7 ± 1684.2	3266.4 ± 1810.9	0.192

N of COVID-19 deaths per 100,000 inhabitants in 2020, mean ± SD	77.4 ± 35.0	92.5 ± 29.0	51.4 ± 38.2	**0.039**[b]
GDP per capita, PPP, 2019 (current international $), mean ± SD	48319.1 ± 19393.9	33554.9 ± 13027.8	43408.8 ± 28713.1	0.211

[a] P-value “Yes” vs “No” = 0.053; “Partly” vs “No” = 0.053; P-value “Yes” vs. “Partly” = 0.97

[b] P-value “Yes” vs “No” = 0.102; “Partly” vs “No” = 0.074; P-value “Yes” vs. “Partly” = 0.343
